# The post-discharge coping difficulty of puerperal women in a middle and low-income tourist city during the COVID-19 pandemic

**DOI:** 10.1186/s12884-023-05554-0

**Published:** 2023-04-13

**Authors:** Yan Liu, Lan-lan Peng, Yi-yuan Zhang, Mei-yin Tang, Mei-qiong Tang, Yun-yun Xu, Zong-ying Ma, Yi Tang, Lingling Gao

**Affiliations:** 1 Affiliated Hospital of Guilin Medical University, Guilin Medical University, Guilin, Guangxi China; 2 School of Nursing, Sun Yat-Sen University, Guangzhou, Guangdong China

**Keywords:** Coronavirus disease 2019, Puerperal woman, Post-discharge, Coping difficulty, Maternal and child health literacy, Social support

## Abstract

**Background:**

Since the coronavirus disease 2019 (COVID-19) pandemic outbreak, the incidence of mental health problems in perinatal women has been high, and particularly prominent in China which was the first country affected by COVID-19. This paper aims to investigate the current situation and the related factors of maternal coping difficulties after discharge during COVID-19.

**Methods:**

General information questionnaires (the Perinatal Maternal Health Literacy Scale, Postpartum Social Support Scale and Post-Discharge Coping Difficulty Scale-New Mother Form) were used to investigate 226 puerperal women in the third week of puerperium. The influencing factors were analyzed by single factor analysis, correlation and multiple linear regression.

**Results:**

The total score of coping difficulties after discharge was 48.92 ± 12.05. At the third week after delivery, the scores of health literacy and social support were 21.34 ± 5.18 and 47.96 ± 12.71. There were negative correlations among health literacy, social support and coping difficulties after discharge (*r* = -0.34, *r* = -0.38,* P* < 0.001). Primipara, family income, health literacy and social support were the main factors influencing maternal coping difficulties after discharge.

**Conclusion:**

During the COVID-19 pandemic, puerperal women in a low- and middle-income city had moderate coping difficulties after discharge and were affected by many factors. To meet the different needs of parturients and improve their psychological coping ability, medical staff should perform adequate assessment of social resources relevant to parturients and their families when they are discharged, so they can smoothly adapt to the role of mothers.

## Background

Puerperium is the stage from delivery of the fetal placenta to the recovery or near-normal non-pregnant state of all organs (except the mammary gland). Lasting around 42 days (6 weeks) after delivery, it is an important period for maternal recovery and neonatal development [1]. The current trend is to shorten the length of hospital stay for low-risk women. Although the readmission rate of women within 6 weeks after discharge has not changed [2], maternal psychological problems have not received effective attention and continuous care. Compared with developed countries, there are some problems in postpartum nursing in China requires improvement, such as increasing the number of postpartum visits, the attention paid to the psychological emotions of mothers and their spouses and broad access qualifications of visitors, and communication barriers in hospital communities, etc., and maternal psychological needs cannot be met [3, 4]. Especially in the 2–3 weeks of the puerperal period, the problems with physical recovery are common: 82% of the parturients are readmitted to hospital within 20 days after discharge due to late postpartum hemorrhage [5]. In addition, complicated family relationship problems may occur due to newborns. The postpartum depression rate of mothers living with their spouses and parents was 1.38 times that of those who did not live together [6], resulting in certain pressure on maternal psychological coping. Maternal Post-Discharge Coping Difficulty (PDCD) is found, namely the self-perceived difficulty of coping with stress, physical rehabilitation, self-care, perception of obtaining support, and ability to take care of the baby information after discharge [7]. Studies [8, 9] have shown that puerpera have higher coping difficulties after discharge, which affects their physical and mental health.

Most studies on maternal coping difficulties were conducted from the perspective of discharge readiness based on Meles’ transition theory. A study [10] also showed that even though puerpera had a good perceived readiness before discharge, this perception changes after coping with various problems after discharge, and they have a constant demand for knowledge about childcare. It can be seen that maternal coping regulation are a process in which the internal and external environment of an individual is interlinked and constantly changing, so factors should also be based on Lazarus’s theory of stress and coping mode [11]. Maternal coping difficulties after discharge are affected by personal, community and social factors. Individual factors are mainly related to cognitive evaluation, and the key index is maternal and child health literacy including individual health knowledge, skills and beliefs [12]. In addition, postpartum social support is an external factor affecting maternal coping after discharge. Improving social support can meet maternal physiological and psychological needs, reduce the occurrence of postpartum depression, and benefit the relationship between mother and child [13].

It is also necessary to consider the environmental factors of the novel coronavirus pandemic. One study showed that the risk of psychological symptoms in perinatal women increased more than two-fold [14]. In China, which was the first to be affected by COVID-19, most patients with anxiety and depression were postpartum [15]. This especially affected those living in economically underdeveloped areas in central and western China since they are more likely to suffer from depression and have more health needs than in other regions [16, 17]. Tourist cities located in western China, which has a high migration rate, had a high probability of imported COVID-19 cases and a wide range of transmission [18]. The extent of maternal coping difficulties in such areas is unknown. Therefore, this study investigated maternal difficulties after discharge in a low- and middle-income tourist city during the COVID-19 pandemic, to propose methods to ensure the quality of postpartum care. This not only improves maternal physical and mental rehabilitation, but also provides a theoretical basis for exploring maternal continuing care and prevention of their disease.

## Methods

### Study design

Based on Meles’ transition theoretical model and Lazarus’ stress and coping theoretical model, relevant variables were determined in this study. This study was reviewed and approved by the ethics committee of the Affiliated Hospital of Guilin Medical University (approval number 2022qTLL-01), including granting permission for the research use of the maternal medical data. Informed consent was signed by the puerpera before discharge, which informed that the post-discharge survey would be conducted through a third-party platform such as WeChat. The principles of informed consent, respect for research subjects, confidentiality, and benefit to research subjects were strictly observed. The outcome assessors were blinded to the exposure status of parturients.

### Samples

The study collected a convenience sample from women giving birth at the Affiliated Hospital of Guilin Medical University from March to April 2022. The study inclusion criteria were women aged ≥ 20 years old who met the criteria of the 9^th^ edition of the Obstetrics and Gynecology of puerperal women [1]. Exclusion criteria were 1) the inability to communicate verbally or in writing, 2)severe impairment of other vital organs (e.g., diagnosis of heart failure, respiratory failure, kidney failure), 3) meeting the diagnostic criteria for postpartum depression in the 5^th^ edition of the Diagnostic and Statistical Manual of Mental Disorders of the American Psychiatric Association, 4) scoring ≥ 13 on the Edinburgh postnatal depression scale (EPDS) after delivery or 5) severe central nervous system diseases requiring antidepressant or anti-anxiety drugs [19].

Before discharge from hospital, subjects signed an informed consent form and filled out a general information questionnaire (which asked for demographic and maternity data). On the third week of puerperium, subjects were contacted via WeChat, an open user communication platform which can be used for investigation. Participants were asked to complete a Questionnaire Star, which included the Perinatal Maternal Health Literacy Scale, Postpartum Social Support Scale, and the Chinese version of the Post-Discharge Coping Difficulties Scale.

A continuous sample bilateral test was used, α level was 0.05, test power (1 − β) was 0.8, and the mean and standard deviation were obtained from the scores of post-discharge coping difficulties of pregnant women with gestational diabetes [9] in the literature (5.22 ± 0.97). An online tool (http://powerandsamplesize.com/Calculators/) was used to calculate the required sample size. This was found to be 153 cases, and taking the rate of uncompleted questionnaires as 20%, the minimum was calculated at 192 cases. The income of urban employees in Guilin is at a low-medium level. According to the 2021 Guangxi Statistical Yearbook [20], the average wage of urban employees in Guilin in 2020 was 6, 522 yuan, ranking 7^th^ among 14 cities in Guangxi.

### Measures

1. The general information questionnaire included two parts. First, demographic data were collected including age, education level, living place, employment status and family per capita monthly income. The other data collected included prenatal status (number of pregnancies, number of children raised, and any complications), intrapartum (mode of delivery) and postnatal (feeding mode, postnatal weight, admission to intensive care unit).
2. The Chinese version of the Post-Discharge Coping Difficulties Scale-New Mother Form (PDCDS) [9] was used for measuring stress, the body’s recovery and baby care after discharge. There are 10 items in the scale, and items 1–6 were accompanied by corresponding open questions. Each item was scored from 0 to 10 points, while items 8 to 10 are scored as the reverse. The total score of the scale ranges from 0 to 80 points. A higher score indicated greater coping difficulty. Participants were divided into a high and low scoring group: the first 27% of the score was the high group, and the last 27% was the low group [21]. The Cronbach’s alpha coefficient, split half reliability and test–retest reliability of the Chinese version of the maternal coping difficulties scale after discharge were 0.97, 0.96 and 0.97, respectively, which were used to measure the maternal coping difficulties. Cronbach’s alpha coefficient of the scale was 0.76.
3. A previous scale [22] of women’s health 28 weeks postpartum was revised after three rounds of expert enquiry to include postpartum maternal and child health literacy content. It covered 27 entries over three dimensions: basic knowledge and concepts, healthy lifestyle and behavior, and basic skills. Each item was scored as “ yes” (scored 1 point), “ no” or “ don’t know” (scored 0 points). According to the standard of Chinese residents’ basic health literacy [23], 80% of the questions can be answered correctly. This is taken as the criterion of this study: a score ≥ 22.4 points is considered as having basic health literacy. The Cronbach’s alpha coefficient of this questionnaire was 0.86, the total split half reliability was 0.78, the Content Validity Index (CVI) was 0.94, and the Spearman correlation coefficient of test–retest reliability was 0.43, indicating good reliability and validity.
4. The postpartum social support scale for Chinese women [24] includes four dimensions (emotion, information, material support, and support assessment), with five items for each dimension. A Likert 4-level scoring method was used to score each item from 0 to 3 (never, rarely, sometimes, usually) and the total score was 0–60. Higher scores indicated more social support after giving birth. The internal consistency of the scale was 0.89 and the content validity was 0.90. The Cronbach’s alpha of this questionnaire was 0.94, and the total split half reliability was 0.80.

### Statistical analyses

SPSS version 25 (IBM® SPSS® Statistics) was used for statistical analysis and inference, and a two-sided test was used. The test level α was 0.05, and *P* < 0.05 was considered statistically significant. Frequency and percentage were used to describe the general information. The measurement data of maternal and infant health literacy, postpartum social support, and coping with difficult situations after discharge were described by mean ± standard deviation (SD). Categorical data were compared by Chi-square (*χ*^*2*^) test. A Fisher’s exact test was performed for data < 1. In univariate analysis, normality and homogeneity of variance of outcome variables (coping difficulty score) should be tested first. When these were met, a t-test was used to compare the differences between two groups, and a one-way ANOVA was used to compare the differences between three or more groups. When the continuous variable was not normally distributed, a rank sum test was used. If the variance of the results was not uniform, a Welch test was performed. Pearson correlation was used to describe the relationship between maternal and child health literacy, social support and coping difficulties after discharge if it was normal, and Spearman correlation analysis otherwise. To avoid confounding factors affecting independent variables, a stepwise regression method was used in multiple linear regression analysis to remove insignificant variables from the equation one by one, to obtain the optimal equation.

### Quality control

To ensure that the puerpera could be contacted after discharge, informed consent and two or more pieces of contact information were obtained from them and their family members during hospitalization. The data were collected with Questionnaire Star through WeChat. Each WeChat link could only be used once and questionnaires with an answer time < 300 s were excluded to ensure authenticity. Two researchers separately checked the data and input it into Excel, crossed-checked for errors, then checked for omissions and performed logical verification to ensure integrity and accuracy.

## Results

### Participation in the survey

**Fig. 1:**
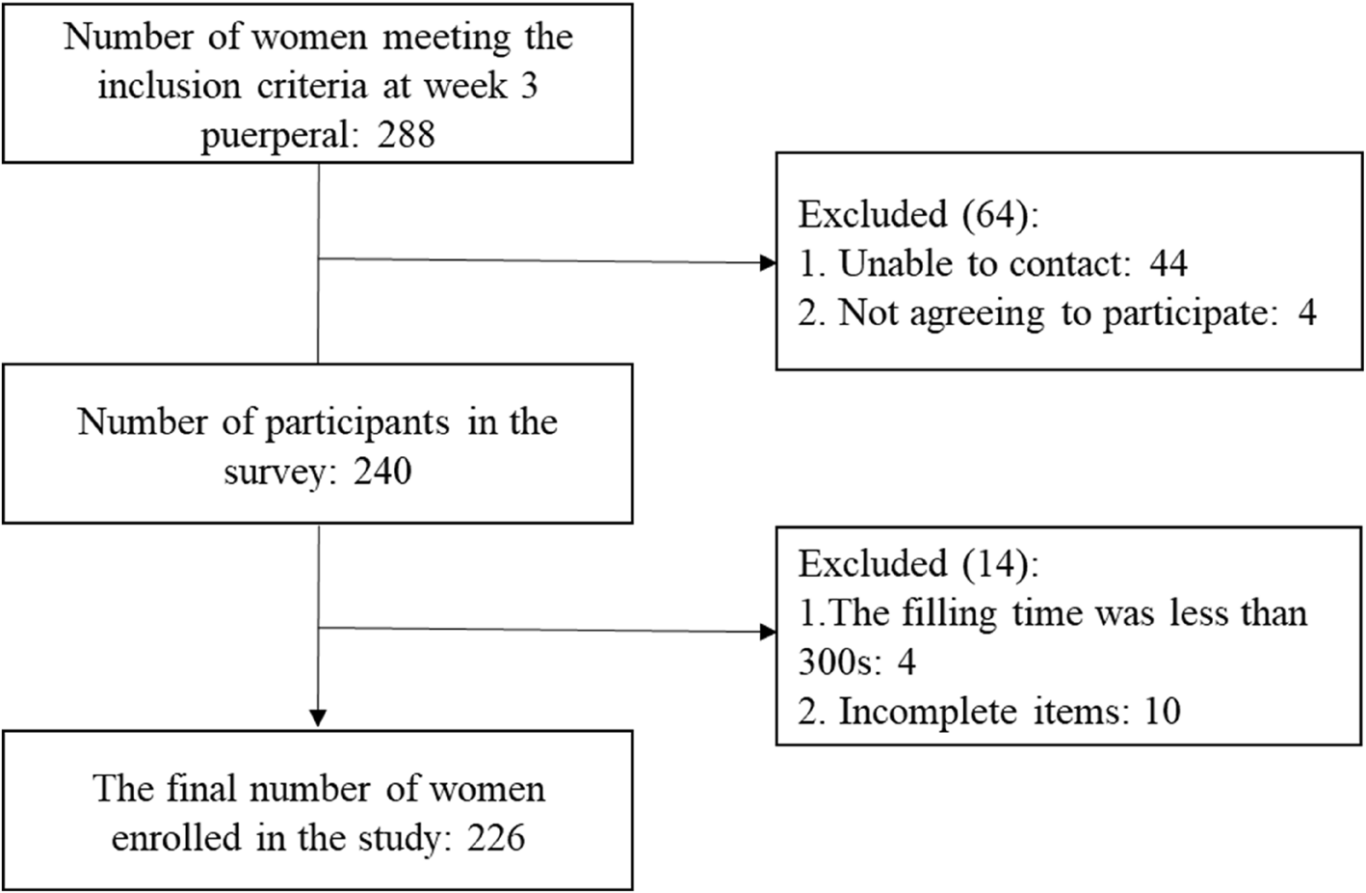
Flow diagram of the study participants

A total of 288 inpatients who met the inclusion criteria were enrolled in the Obstetrics Department of ahospital in Xiufeng District of Guilin City from March to April 2022. Of these patients, 44 mothers and their families could not be contacted, and 4 mothers reported they were busy with their newborns and could not take part in the test. Therefore, 240 questionnaires were recovered (a response rate of 83.33%), and 226 valid questionnaires were recovered (an effective response rate of 78.47%). See Fig. 1 can be seen for specific exclusions.

### Basic maternal characteristics

A total of 226 parturients with an average age of 31.85 ± 4.25 years (range, 21–42 years) were included in the study, and of these 63.3% had junior college/bachelor’s degree. Most (89.4%) lived in cities and towns. The average monthly income of 58.4% of mothers was < 5,000 yuan, 26.5% between 5,000—8,000 yuan, and 15.1% > 8,000 yuan. About two thirds (62.4%) were employed or self-employed, 10.2% were farmers and 27.4% were unemployed. Most of the women did not attend the school for pregnant women (83.2%) did not attend school for pregnant women, 55.8% were primiparas, and 44.1% had one or two complications during pregnancy. Nearly half (47.3%) of the women had a vaginal delivery, and the hospital stay days ranged from 2 to 12 days (average hospital days were 4.25 ± 1.62 days), and 60.6% of the women were hospitalized for 2 to 4 days. Most (71.2%) neonates were not admitted to the neonatal care unit, 88.4% were full-term and 60.6% had mixed feeding. After discharge, 56.6% of the mothers’ primary caregivers during the follow-up period were their spouses, and the average number of follow-up visits was 1.3.

### Scores of maternal post-discharge coping difficulty

The total score on the coping difficulties scale after discharge was 48.92 ± 12.05, and the total average score was 4.89 ± 1.20. The scores were divided into 27% by high and low scores. After ranking the scores from low to high, the low score was lower than or equal to 42; after ranking the scores from high to low, the high score was more than or equal to 55 points. The score ranges from 41 to 54, which is in the middle group. The score of this study is in the middle level, and the score of each item is shown in Table 1.

**Table 1:** Scores of maternal post-discharge coping difficulty (*n* = 226, M ± SD)

Maternal post-discharge coping difficulties items	Average score
**Total score**	4.89 ± 1.20
1. How stressful has your life been?	5.03 ± 2.37
2. How much difficulty have you had with your postpartum recovery?	5.06 ± 2.20
3. How much difficulty had you had with caring for baby?	5.32 ± 1.92
4. How much difficulty had you had feeding your baby?	5.17 ± 2.28
5. How difficult has the time been for your family members or other close people?	4.79 ± 2.22
6. How much help have you needed with caring for baby?	5.45 ± 2.29
7. How much emotional support have you needed?	6.37 ± 2.42
8. How confident have you felt in your ability to care for your own needs since giving birth?	3.57 ± 2.06
9. Have you been able to take care of your baby since giving birth?	4.14 ± 2.12
10. How well have you adjusted to being at home since your baby was born?	3.98 ± 2.05

### Univariate analysis of maternal post-discharge coping difficulty

The differentiation analysis of social demographic and obstetrical characteristics of parturients showed that there were difference significant in education level, family income, employment status, number of births, and coping difficulties of multiple pregnancies after discharge (*P* < 0.05), as shown in Table 2.

**Table 2:** Univariate analysis of maternal post-discharge coping difficulty (*n* = 226, M ± SD)

Variables	N (%)	Post-discharge coping difficulty	*F/t*	*P*
**Age (years)**			0.10	0.902
20–24	7(3.1)	50.85 ± 9.94		
25–34	161(71.2)	48.94 ± 11.90		
≥ 35	58(25.7)	48.65 ± 12.84		
**Educational level**			5.60^a^	0.001
Junior high school education or below	21(9.3)	44.52 ± 10.13		
High school education or technical secondary school education	47(20.8)	54.87 ± 13.23		
College/bachelor’s degree	143(63.3)	47.65 ± 11.37		
Master’s degree or above	15(6.6)	48.66 ± 11.78		
**Residential location**			0.07	0.939
Urban	202(89.4)	48.95 ± 12.10		
Rural	24(10.6)	48.75 ± 11.89		
**Per capita monthly household income (yuan, RMB)**			8.23^a^	0.001
< 5000	132(58.4)	51.56 ± 11.26		
5000–8000	60(26.5)	45.10 ± 13.68		
> 8001	34(15.1)	45.47 ± 9.30		
**Employment status**			15.67^b^	< 0.001
Employed	141(62.4)	47.68 ± 11.90		
Farmers/private individuals	23(10.2)	40.52 ± 11.28		
Unemployed	62(27.4)	54.87 ± 9.94		
**Maternity school**			1.94	0.053
Not attended	188(83.2)	49.62 ± 12.23		
Participated	38(16.8)	45.47 ± 10.64		
**Delivery times**			4.02^b^	< 0.001
Unipara primipara	126(55.8)	51.68 ± 12.14		
Pluripara	100(44.2)	45.46 ± 11.07		
**Number of prenatal comorbidities (category)**			0.16	0.845
None	88(38.9)	48.50 ± 12.38		
1–2	125(55.3)	49.07 ± 11.43		
≥ 3	13(5.8)	50.46 ± 16.07		
**Multiple births**			0.29^a^	0.020
Yes	7(3.1)	55.85 ± 6.06		
No	219(96.9)	48.70 ± 12.14		
**Delivery method**			0.62	0.536
Vaginal delivery	107(47.3)	48.28 ± 11.68		
Cesarean delivery	87(38.5)	48.96 ± 13.44		
Midwifery (forceps / lateral episiotomy)	32(14.2)	51.00 ± 8.98		
**Number of postpartum complications (category)**			0.55	0.575
None	133(58.9)	49.25 ± 11.58		
1	78(34.5)	47.94 ± 11.96		
≥ 2	15(6.6)	51.13 ± 16.51		
**Hospitalization days**			0.52	0.593
2–4	137(60.6)	49.13 ± 11.03		
5–7	79(35.0)	48.16 ± 13.75		
≥ 8	10(4.4)	52.10 ± 11.68		
**Main caregivers after discharge**			0.40	0.753
Spouse	128(56.6)	49.57 ± 12.74		
Parents	21(9.3)	46.71 ± 13.83		
Parent-in-law	14(6.2)	48.14 ± 7.72		
Maternity centers or matrons	63(27.9)	48.52 ± 10.86		
**Neonatal intensive care unit**			1.76	0.079
Yes	65(28.8)	51.04 ± 11.02		
No	161(71.2)	48.07 ± 12.38		
**Gender of newborn (7 sets of twins)**			0.13	0.894
Female	118(50.6)	49.03 ± 11.74		
Male	115(49.4)	48.81 ± 12.43		
**Newborn birth weight (g)**			0.18	0.830
< 2500	20(8.6)	48.35 ± 9.59		
2500 g-4000	206(88.4)	49.07 ± 12.40		
> 4000	7(3.0)	46.42 ± 8.77		
**Feeding mode after discharge**			0.72	0.488
Artificial feeding	14(6.2)	45.92 ± 11.24		
Breastfeeding	75(33.2)	48.32 ± 10.90		
Mixed feeding	137(60.6)	49.56 ± 12.73		
**Home visits (times)**			0.92	0.400
None	3(1.3)	57.00 ± 10.44		
1	191(84.5)	48.59 ± 12.08		
≥ 2	32(14.2)	50.18 ± 11.98		

^a^At the 0.05 level (two-tailed), the association was significant

^b^Significant association at the 0.01 level (two-tailed)

### Correlation between post-discharge coping difficulty, health literacy and social support at discharge 3 weeks after delivery

The score of health literacy was 21.34 ± 5.18 and the score of social support was 47.96 ± 12.71, both of which were at a medium level and negatively correlated with coping difficulties after discharge (Table 3).

**Table 3:** Correlation between post-discharge coping difficulty, health literacy and social support at discharge 3 weeks after delivery

Variables	Dimensionalities	Post-discharge coping difficulty	
		***r***	***P***
**Health literacy**	Total score	-0.34	< 0.001
	Basic knowledge and ideas	-0.26	< 0.001
	Health lifestyle and behavior	-0.33	< 0.001
	Basic skills	-0.32	< 0.001
**Social support**	Total score	-0.38	< 0.001
	Emotional support	-0.24	< 0.001
	Material support	-0.24	< 0.001
	Information support	-0.42	< 0.001
	Assessment of support	-0.37	< 0.001

### Multiple linear regression analysis of maternal post-discharge coping difficulty

The significant variables in univariate and correlation analysis were used as independent variables, and the scores of maternal coping difficulties after discharge were used as dependent variables to conduct multiple linear regression analysis. As continuous variables, health literacy, social support score and difficulty coping score were directly input into the original data. Other variables were assigned respectively. The Durbin-Watson value was 2.29, indicating a high probability that the observations were independent. The regression model was statistically significant in general (*P* < 0.001), the *R* value was 0.55, and the *R*^*2*^ value was 0.30. Regression equation: coping difficulties after discharge = 79.33–5.65 × primipara—2.55 × monthly family income—0.42 × health literacy—0.31 × social support. Primipara, family income, health literacy and social support were the main influencing factors of maternal coping difficulties after discharge, as shown in Table 4.

**Table 4:** Multiple linear regression analysis of maternal post-discharge coping difficulty

Variables	Unstandardized coefficients		Standardized Coefficients	*t*	*p*
	**B**	**Standard error**	**Beta**		
Constant term	79.33	3.56		22.28	< 0.001
Social support	-0.31	0.05	-0.32	-5.49	< 0.001
Delivery times	-5.65	1.43	-0.23	-3.95	< 0.001
Health literacy	-0.42	0.14	-0.18	-2.96	0.003
Per capita monthly household income	-2.55	0.94	-0.15	-2.70	0.007

*R* = 0.553, *R*^*2*^ = 306, adjusted *R*^*2*^ = 285, *F* = 21.972, *P* < 0.001

## Discussion

The total score of maternal coping difficulties after discharge was 48.92 ± 12.05, and the total average score was (4.89 ± 1.20), showing a moderate degree of difficulty, but higher than a previous study (2.2 ± 1.30, [8]). The reason was mainly related to the distribution of different ethnic groups. In this study, the proportion of primiparas (55.8%) was higher than that in Weiss and Lokken (39.4%) [8]. However, the total mean score of coping difficulties after discharge and needing emotional support in the third week after delivery (6.37 ± 2.42) were lower than the overall result of coping difficulties after discharge and the score of emotional needs in a previous survey of women with gestational diabetes mellitus (GDM) [9]. The analysis mainly showed that pregnant women with GDM would pay more attention to the risks of perinatal diseases, health management under the disease state, and higher pressure to cope with and emotional needs to deal with potential problems caused by complications during pregnancy. In contrast to previous studies, this study found that maternal psychological stressors included not only parenting stress and interpersonal relationships, but also the current social environment stress caused by the COVID-19 pandemic. The number of both maternal caregivers and visits made were limited during hospitalization, and postpartum face-to-face communication opportunities with medical staff and friends were reduced.. It is therefore not surprising that the incidence of maternal mental health problems was substantially higher than before the pandemic [25]. The greater the perceived stress, the greater increase in brain neural activity, the higher the risk of negative emotions (anxiety and depression) [25, 26], and the more emotional support the parturient needed. Therefore, support should also be offered to women after childbirth, not only during pregnancy or for maternal complications.

For postpartum health care after discharge, most cities carried out visits according to China’s national maternal healthcare standards [27] (postpartum visit time should be in postpartum within 7 days, 28 days, all at a time). The postpartum visit rate increased year by year, but less to the evaluation of maternal psychological needs. According to the China Health Statistical Yearbook 2020 [28], the rate of postpartum visits in Guangxi was 95.7% in 2019, higher than the national average of 94.1%, and the visits met the basic requirements. In this study, the average number of family visits was 1.3; most parturients (84.5%) got one family visit, 14.2% got more than two family visits, and only 1.3% did not get family visit. However, most postpartum visits were within one week after discharge, which means that the needs of parturients in the third week after delivery were not met. In addition, the content of visits did not focus on the psychological emotions of parturients and their spouses, thus the psychological coping problems of parturients were not alleviated [29]. Nearly half (47%) [30] stated that they discussed their physical and mental health for less than three minutes, or there was no discussion at all, and most of the time was spent with the baby. Since the current supervision mechanism of maternal care centers is not perfect, the puerpera who choose these centers or matrons still face difficulties in coping after discharge. The third week after delivery, when women especially face prominent problems, and cope with difficult situations, is still a critical period where healthcare must improve. Therefore, mothers’ health could be improved by establishing effective communication (e.g. a midwife-mediated WeChat follow-up group, consulting platform, etc.), focusing on the mothers’ dynamic needs. This should be followed by health guidance and advice, which will help mothers to improve and maintain their mental and physical health and mental recovery.

Univariate and multivariate regression analysis showed that family income, primipara, social support and health literacy were the main influencing factors of maternal coping difficulties after discharge. Mothers with a monthly family income < 5000 yuan had a coping difficulties score of 51.56 ± 11.26, which was the middle group. Similar to previous results [31], those with a higher family income were more likely to go to the maternity center or hire matrons to help with postpartum care, and they also had lower coping difficulties. During the COVID-19 pandemic, the global economy has been severely affected. Social distancing and home isolation have reduced social activities and led to mass unemployment in different industries, which has had a certain impact on household income, especially in tourist-oriented cities. In addition, due to the opening of the two- and three-child policy in China, the child-rearing cost of families is also increasing, which increases the economic and psychological burden of mothers with lower family income. Especially for premature infants and newborns discharged from intensive care units, the costs of rehabilitation and care will continue long after discharge.

The score of coping difficulties of primiparas was 51.68 ± 12.14, higher than in multiparas and similar to primiparas with GDM after discharge [31]. Delivery and postnatal experience often can cause physical and emotional changes to the mother, who faces maternal postpartum uterus rehabilitation and recovery problems such as wound healing, infant feeding and anomalies, dealing with family relationships and other pressure sources. This is especially true for first-time mothers who lack experience and may feel unable to deal with these questions, and therefore may experience psychological problems. In recent years, the psychological problems of pregnant women have gradually attracted the attention of medical and health workers. After 2011, the government successively introduced corresponding policies, and industry experts have regularly updated theories and formulated consensus guidelines on the management of maternal mental health [29]. Maternal health care has been standardized in various medical institutions, and the rate of postpartum visits has also improved. However, the psychological problems of puerpera have not received effective and comprehensive attention, and the quality of postpartum visits needs to be further improved. The overwhelming majority of mothers think follow-up after discharge was too simple: professionals only focused on baby care, ignored the emotional needs of parturients and their spouses and failed to meet the postpartum care needs of primiparas; this resulted in a large gap between the expectation and perception of puerperium health management [4].

The score of social support of puerperal women after discharge in the third week was 47.96 ± 12.71, which was similar to the score of 41.87 ± 6.75 in a previous survey on the level of social support of postpartum women at 4–6 weeks [32]. In the correlation analysis, social support after discharge was negatively correlated with coping difficulties. In the multivariate linear analysis, social support after discharge affected the coping situation. The higher the maternal social support after discharge, such as emotional, information and material support, the less the coping difficulties. Social support has a positive effect on puerperium body recovery and psychological pressure, and is an important factor to avoid postpartum depression. Especially in the social environment of the COVID-19 pandemic, information support has the greatest impact on puerpera. Although puerpera obtain relevant health information from online platforms or remote nursing, different platforms provide different information, which requires puerpera to have a certain judgment ability, and can fail to meet their needs for timely feedback and help. In addition, social isolation measures also reduced the opportunities for information exchange among community peers, so that puerperal women felt that their information needs for professional care were not met.

As previously stated [33], during the COVID-19 pandemic attention should be paid to the social support of pregnant women. Before discharge, families should be investigated to evaluate the effect of education, and the social support system that encourages the participation of spouses should be used to help puerperal women make a smooth transition. In this study, social support after discharge was negatively correlated with coping difficulties, which further confirmed the importance of continuous systematic support and guidance for puerpera, regular remote social support services for puerpera and their families, and safe face-to-face home care services. These are all important directions for subsequent research.

The score of maternal and infant health literacy at the third postpartum week was medium. After the construction of a baby-friendly hospital, pregnant and perinatal women have gained more knowledge of maternal and infant health care through medical institutions. However, puerperal women did not fully use their knowledge to improve infant health in home care, especially primipara who had lower maternal and child health literacy, self-care ability and parenting confidence, and failed to respond to difficulty [34]. As previously suggested [35], the maternal health literacy level of pregnant women should be assessed during prenatal care; and specific, measurable, achievable and reasonable lifestyle goals jointly formulated with the puerpera. Alongside pregnant women and their partners, health professionals and community doctors, midwives, nurses, social workers, and psychologists should collaborate to integrate health into the whole life cycle of different forms of care. This would allow targets to be tracked in postpartum follow-up. Improving maternal health literacy would also reduce the degree of postpartum coping difficulties.

### Strengths and limitations

To the best of our knowledge, this is the first study to investigate how mothers in a low—and middle-income tourist city in China are coping with difficult post-discharge situations during the COVID-19 pandemic. COVID-19 has increased global mental health issues, and it is important to understand what needs to be addressed in post-natal care for women at home during this pandemic. Medical and health care personnel can put forward solutions and methods for ensuring the quality of postpartum care, to promote physical and mental rehabilitation of mothers. However, this study took place in one hospital and the sample size might be subject to sampling error. In addition, because the survey was self-administered online, misunderstanding of the questions by participants could not be ruled out. Since maternal child-rearing costs were not measured, subsequent studies can evaluate the impact on puerperium coping from the perspective of economic costs.

## Conclusion

Against the background of the COVID-19 pandemic, this study investigated the current situation of maternal discharge difficulties in a hospital in Guilin City, a famous tourist city. Women in the third week of puerperium had a moderate degree of difficulty. Low health literacy and social support at the third week of puerperium, low family income, low education level and primiparas were the main influencing factors. Women living in underdeveloped tourist cities in western China face more economic pressure. Medical personnel in the hospital should collaborate with mothers and their families to provide adequate social resource assessment, links, social workers and community resources for women, especially those without professional and social support. This would satisfy the demands of mothers and improve their coping ability to deal with problems in home care, and successfully transition into motherhood.

## Data Availability

The datasets generated and analyzed during the current study are available from the corresponding author upon reasonable request.

## Abbreviations

COVID-19: Coronavirus disease 2019
PDCD: Post-discharge coping difficulties
GDM: Gestational Diabetes Mellitus
SD: Standard deviation
VIF: Variance inflation factor
